# Supplementary material for: Integrated Analysis of miRNA-mRNA Network Reveals Different Regulatory Patterns in the Endometrium of Meishan and Duroc Sows during Mid-Late Gestation
Source: Animals (Basel). 2020 Mar 3;10(3):420. doi: 10.3390/ani10030420 (PMC7143271; doi:10.3390/ani10030420)
Supplement: Supplementary file 1 [file animals-10-00420-s001.zip › Supplementary Materials/Figure S1 Crucial pathways were clustered from DE miRNAs target genes.docx]

**
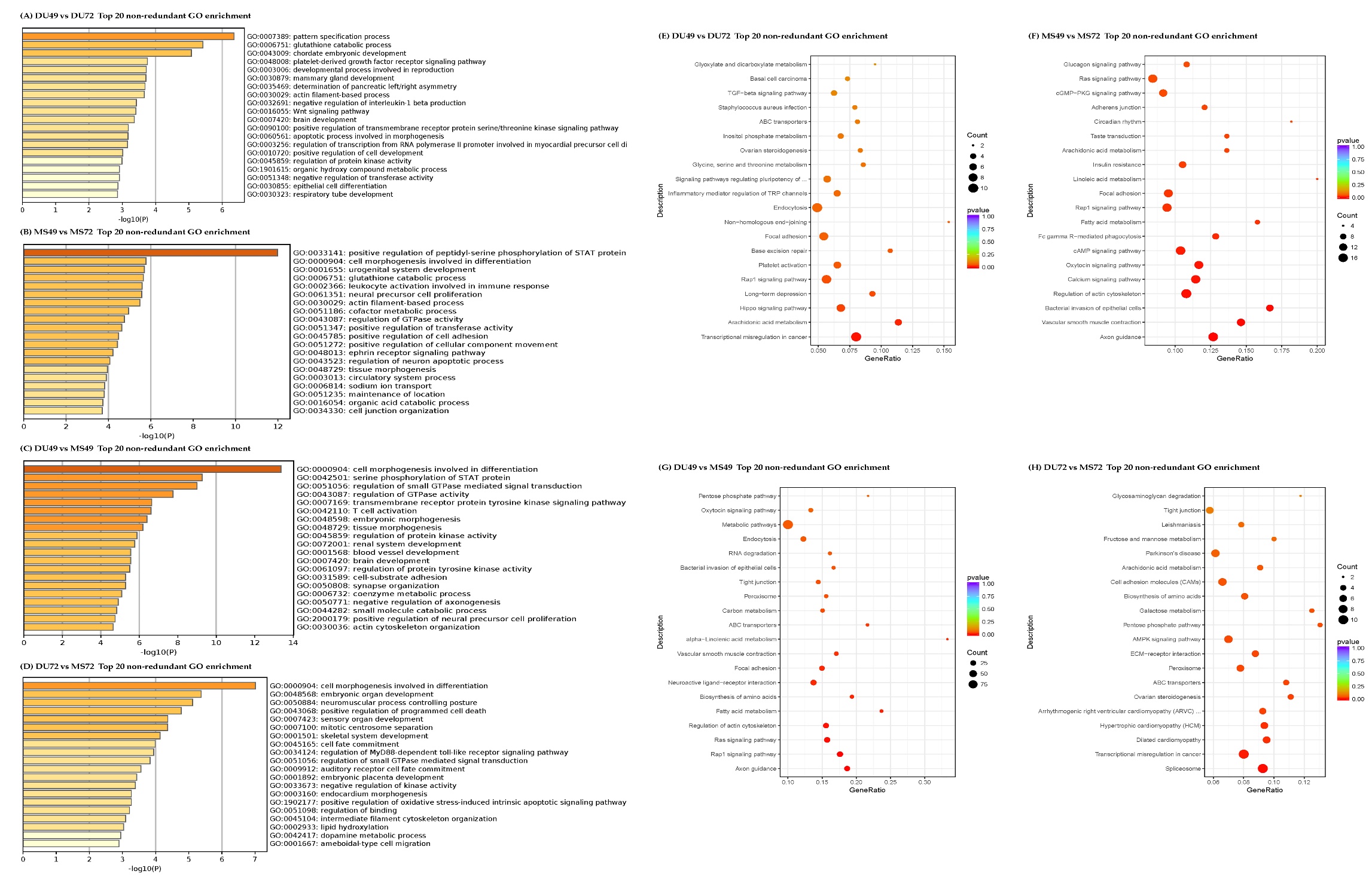
**

**Figure S1.** Crucial pathways were clustered from DE miRNAs target genes. Top 20 non-redundant GO enrichment (**A, B, C, D**) and top 20 of KEGG pathway enrichment (**E**, **F, G, H**) clustered by DE miRNAs target genes, respectively.
