# Supplementary material for: Integrated Analysis of miRNA-mRNA Network Reveals Different Regulatory Patterns in the Endometrium of Meishan and Duroc Sows during Mid-Late Gestation
Source: Animals (Basel). 2020 Mar 3;10(3):420. doi: 10.3390/ani10030420 (PMC7143271; doi:10.3390/ani10030420)
Supplement: Supplementary file 1 [file animals-10-00420-s001.zip › Supplementary Materials/Table S1 PCR primers for qRT-PCR validation of differentially expressed genes and miRNAs between Meishan and Duroc sows at GD49 and GD72.docx]

**Table S1.** PCR primers for qRT-PCR validation of differentially expressed genes and miRNAs between Meishan and Duroc at GD49 and GD72.

| Genes | Gene name | Accession NO. | Forward primer sequence (5’ to 3’) | Reverse primer Sequence (5’ to 3’) | Amplicon (bp) | Tm (°C) |
| --- | --- | --- | --- | --- | --- | --- |
|  | *NPTX1* | XM_003131134.5 | GAAACGACACGGAGGAAAGA | CGGGCAGGCTCTTCTTCA | 171 | 58 |
|  | *ESR1* | XM_021083061.1 | TTGCTGGCTACATCATCTCG | CACGGTGGATATGGTCCTTC | 193 | 58 |
|  | *APLN* | XM_003360446.4 | CGTTCGCTGCTCGTCTG | CAGGGTCTCCCAAGTCAGG | 114 | 60 |
|  | *IHH* | XM_021074681.1 | GCTCACCCCTAACTACAATCCC | TCCTCGTCCCAGCCCTCGGTCA | 168 | 60 |
| genes | *EGR1* | XM_003123974.6 | CCATGATCCCTGACTATCTGT | GGACTGGTAGGTGGTGTTGAG | 191 | 59 |
|  | *CDHR2* | XM_013994874.2 | TGAGCGACAATCACAATAACCC | TCCACAGCCAGCACAGAAAA | 157 | 60 |
|  | *EGF* | NM_214020.2 | ATCTCAGGAATGGGAGTCAACC | TCACTGGAGGATGGAATACAGC | 165 | 60 |
|  | *β-actin* | XM_003124280.5 | GCCAACCGTGAGAAGATGACT | GTGACCCCATCCCCAGAGT | 140 | 60 |
|  | ssc-miR-671-5p | MIMAT0025381 | TAGGAAGCCCTGGAGGGG | - | - | 59 |
|  | ssc-miR-30c-3p | MIMAT0022922 | GCTGGGAGAAGGCTGTTTACTCT | - | - | 60 |
| miRNAs | ssc-miR-503 | MIMAT0010189 | TAGCAGCGGGAACAGTACTGC | - | - | 60 |
|  | ssc-miR-452 | MIMAT0025374 | GGAACTGTTTGCAGAGGAAACTG | - | - | 59 |
|  | ssc-miR-9-1 | MIMAT0002168 | GGGGTCTTTGGTTATCTAGCTGTATG | - | - | 60 |
|  | ssc-miR-23a | MIMAT0002133 | TCATCACATTGCCAGGGATTT | - | - | 59 |
|  | ssc-miR-19b | MIMAT0013950 | AGGTGGGTGTGCAAATCCAT | - | - | 59 |
