# Supplementary material for: Integrated Analysis of miRNA-mRNA Network Reveals Different Regulatory Patterns in the Endometrium of Meishan and Duroc Sows during Mid-Late Gestation
Source: Animals (Basel). 2020 Mar 3;10(3):420. doi: 10.3390/ani10030420 (PMC7143271; doi:10.3390/ani10030420)
Supplement: Supplementary file 1 [file animals-10-00420-s001.zip › Supplementary Materials/Table S2 Sequences of primers used for amplification of wild type EGF CDS region and ESR1 3’UTR region.docx]

**Table S2.** Sequences of primers used for amplification of wild type *EGF* CDS region and *ESR1* 3’UTR region.

| Gene name | Accession NO. | Forward primer sequence (5’ to 3’) |
| --- | --- | --- |
| EGF-CDS-F | NM_214020.2 | TAACTCGAGCACCTTACCCAGAAGCAGA |
| EGF-CDS-R |  | TAAAGCGGCCGCGTAGTTTCCCTCCGTATTTG |
| ESR1-3’UTR-F | XM_021083061.1 | TAACTCGAGTGAGGGAGAAAGGGAAAG |
| ESR1-3’UTR-R |  | TAAAGCGGCCGCCCTGCGAAATCGGAAA |
