# Supplementary material for: Integrated Analysis of miRNA-mRNA Network Reveals Different Regulatory Patterns in the Endometrium of Meishan and Duroc Sows during Mid-Late Gestation
Source: Animals (Basel). 2020 Mar 3;10(3):420. doi: 10.3390/ani10030420 (PMC7143271; doi:10.3390/ani10030420)
Supplement: Supplementary file 1 [file animals-10-00420-s001.zip › Supplementary Materials/Table S3. The basic statistics for RNA-seq reads generated from endometrium tissues of 12 sows with Meishan and Duroc during the mid-late gestation.docx]

**Table S3**. The basic statistics for RNA-seq reads generated from endometrium tissues of 12 sows with Meishan and Duroc during the mid-late gestation.

| Sample name^a^ | Raw reads | Clean reads | Error rate(%) | Q20^b^(%) | Q30^c^(%) | Total Mapped Reads | Uniquely Mapped Reads |
| --- | --- | --- | --- | --- | --- | --- | --- |
| DU49_1 | 51285702 | 48756280 | 0.02 | 96.22 | 91.47 | 44497805(91.27%) | 43146674(88.49%) |
| DU49_2 | 61509074 | 58468382 | 0.02 | 96.25 | 91.52 | 53506197(91.51%) | 51714781(88.45%) |
| DU49_3 | 60499254 | 57396038 | 0.02 | 96.19 | 91.41 | 52547614(91.55%) | 50295494(87.63%) |
| DU72_1 | 54655492 | 51654876 | 0.02 | 95.52 | 89.88 | 46428127(89.88%) | 44923622(86.97%) |
| DU72_2 | 63785184 | 62163268 | 0.01 | 97.21 | 93.17 | 57954655(93.23%) | 55928301(89.97%) |
| DU72_3 | 61395904 | 58096414 | 0.02 | 95.74 | 90.26 | 52528886(90.42%) | 50948728(87.70%) |
| MS49_1 | 54132668 | 49667426 | 0.02 | 95.65 | 89.98 | 44396660(89.39%) | 43005706(86.59%) |
| MS49_2 | 59705380 | 55226030 | 0.02 | 96.03 | 90.64 | 49880201(90.32%) | 48244933(87.36%) |
| MS49_3 | 53060196 | 49005000 | 0.02 | 96.16 | 90.89 | 44544404(90.90%) | 42863566(87.47%) |
| MS72_1 | 56966806 | 52106470 | 0.02 | 95.76 | 90.29 | 46242727(88.75%) | 44737426(85.86%) |
| MS72_2 | 63769996 | 58948300 | 0.02 | 96.16 | 90.95 | 53186037(90.22%) | 51605535(87.54%) |
| MS72_3 | 66252936 | 61190596 | 0.02 | 96.21 | 91.04 | 55085334(90.02%) | 52270461(85.42%) |

Note: a MS means Meishan pig, DU means Duroc pig, 49 and 72 mean 49 day’s pregnancy and 72 day’s pregnancy, DU49_1, DU49_2 and DU49_3 refer to the three biological replicates sows in DU49 group, the rest of groups are with same name rules.

b Q20: the proportion of bases with a phred base quality score greater than 20; i.e., the proportion of read bases whose error rate is less than 1%.

c Q30: the proportion of bases with a phred base quality score greater than 30; i.e., the proportion of read bases whose error rate is less than 0.1%.
