# Supplementary material for: Integrated Analysis of miRNA-mRNA Network Reveals Different Regulatory Patterns in the Endometrium of Meishan and Duroc Sows during Mid-Late Gestation
Source: Animals (Basel). 2020 Mar 3;10(3):420. doi: 10.3390/ani10030420 (PMC7143271; doi:10.3390/ani10030420)
Supplement: Supplementary file 1 [file animals-10-00420-s001.zip › Supplementary Materials/Table S4 Distribution of clean reads in the pig genome.docx]

**Table S4.** Distribution of clean reads in the pig genome.

| Sample name | exons (%) | Intron (%) | Intergenic (%) |
| --- | --- | --- | --- |
| DU49_1 | 84.13 | 9.63 | 6.24 |
| DU49_2 | 85.12 | 9.32 | 5.56 |
| DU49_3 | 89.32 | 6.40 | 4.28 |
| DU72_1 | 85.36 | 9.57 | 5.07 |
| DU72_2 | 86.11 | 8.72 | 5.18 |
| DU72_3 | 87.04 | 7.89 | 5.07 |
| MS49_1 | 87.12 | 7.53 | 5.35 |
| MS49_2 | 86.43 | 8.22 | 5.35 |
| MS49_3 | 87.75 | 7.36 | 4.89 |
| MS72_1 | 86.23 | 8.22 | 5.54 |
| MS72_2 | 87.61 | 6.92 | 5.46 |
| MS72_3 | 90.84 | 5.16 | 4.00 |
