# Supplementary material for: Integrated Analysis of miRNA-mRNA Network Reveals Different Regulatory Patterns in the Endometrium of Meishan and Duroc Sows during Mid-Late Gestation
Source: Animals (Basel). 2020 Mar 3;10(3):420. doi: 10.3390/ani10030420 (PMC7143271; doi:10.3390/ani10030420)
Supplement: Supplementary file 1 [file animals-10-00420-s001.zip › Supplementary Materials/Table S5. The basic statistics for miRNA-seq reads generated from endometrium tissues of 12 sows with Meishan and Duroc during the mid-late gestation.docx]

**Table S5**. The basic statistics for miRNA-seq reads generated from endometrium tissues of 12 sows with Meishan and Duroc during the mid-late gestation.

| Sample name^a^ | Raw reads | Clean reads | Error rate(%) | Q20^b^(%) | Q30^c^(%) | Total sRNA | Mapped sRNA |
| --- | --- | --- | --- | --- | --- | --- | --- |
| DU49_1 | 13125752 | 12845459 | 0.01 | 98.78 | 97.55 | 12619906 | 10980674 |
| DU49_2 | 22858944 | 21610577 | 0.01 | 99.61 | 98.80 | 19203438 | 17818253 |
| DU49_3 | 16495762 | 16203428 | 0.00 | 99.81 | 99.43 | 15666158 | 15090210 |
| DU72_1 | 18249860 | 17804522 | 0.01 | 98.43 | 96.79 | 17326676 | 15634368 |
| DU72_2 | 14792491 | 14635238 | 0.00 | 99.80 | 99.41 | 14271465 | 13943460 |
| DU72_3 | 13615771 | 13328553 | 0.01 | 99.69 | 99.05 | 12871854 | 12273215 |
| MS49_1 | 12637289 | 12433667 | 0.00 | 99.82 | 99.44 | 11917885 | 11313774 |
| MS49_2 | 16888884 | 16572967 | 0.01 | 98.57 | 96.98 | 16004393 | 14108820 |
| MS49_3 | 15781329 | 15226158 | 0.00 | 99.80 | 99.40 | 14588755 | 14076702 |
| MS72_1 | 16154248 | 15864484 | 0.01 | 98.81 | 97.58 | 15495437 | 13790387 |
| MS72_2 | 11642745 | 11504963 | 0.01 | 99.76 | 99.25 | 11107802 | 10645252 |
| MS72_3 | 16557568 | 16373799 | 0.01 | 99.64 | 98.89 | 15896649 | 15409728 |

Note: a MS means Meishan pig, DU means Duroc pig, 49 and 72 mean 49 day’s pregnancy and 72 day’s pregnancy, DU49_1, DU49_2 and DU49_3 refer to the three biological replicates sows in DU49 group, the rest of groups are with same name rules.

b Q20: the proportion of bases with a phred base quality score greater than 20; i.e., the proportion of read bases whose error rate is less than 1%.

c Q30: the proportion of bases with a phred base quality score greater than 30; i.e., the proportion of read bases whose error rate is less than 0.1%.
